# An enhanced staining method K-B-2R staining for three-dimensional nerve reconstruction

**DOI:** 10.1186/s12868-019-0515-7

**Published:** 2019-07-08

**Authors:** Peng Luo, Jianghui Dong, Jian Qi, Yi Zhang, Xiaolin Liu, Yingchun Zhong, Cory J. Xian, Liping Wang

**Affiliations:** 1 Department of Bone and Joint Surgery, Shenzhen Sixth People’s Hospital, Shenzhen, 518000 Guangdong China; 2 Department of Hand Surgery, Ningbo No. 6 Hospital, Ningbo, 315040 China; 3 School of Pharmacy and Medical Sciences, and UniSA Cancer Research Institute, University of South Australia, Adelaide, SA 5001 Australia; 4 Department of Orthopedics Trauma and Microsurgery, The First Affiliated Hospital of Sun Yat-sen University, Guangzhou, 510080 Guangdong China; 5 Department of Plastic and Reconstructive Surgery, The First Affiliated Hospital of Sun Yat-sen University, Guangzhou, 510080 Guangdong China; 6 School of Automation, Guangdong University of Technology, Guangzhou, 510006 China

**Keywords:** K-B-2R, Peripheral nerves, Three-dimensional reconstruction, Acetylcholinesterase staining, Fascicle texture feature, Image segmentation

## Abstract

**Background:**

Three-dimensional (3D) reconstruction of human peripheral nerves, as a useful tool to understand the nerve internal information and functional basis, has become an important area of research in the peripheral nerve field.

**Methods:**

In this study, we proposed a two-dimensional (2D) Karnovsky–Roots toluidine blue ponceau 2R (K-B-2R) staining method based upon conventional Karnovsky–Roots staining. It significantly improved the ability to display nerve fascicles, motor and sensory nerve fiber textures. In this method, Karnovsky–Roots staining was carried out, followed by toluidine blue counterstain and ponceau 2R counterstain.

**Results:**

Comparisons were conducted between the three methods in staining of median nerve sections, which showed similar distribution characters in acetylcholinesterase-positive sites. The additional counterstaining did not change the basis of Karnovsky–Roots staining. However, the resulting images from this new method significantly facilitated the subsequent 3D nerve reconstruction and 3D printing.

**Conclusions:**

These results show that the new staining method significantly enhanced the display qualities of nerve fascicle edges and fiber textures of motor and sensory nerves and facilitated 3D nerve reconstruction.

## Background

Peripheral nerve is an important organ tissue that mainly functions to receive afferent sensory impulses and send out efferent motor instructions. As such, the peripheral nervous system has been studied extensively both macroscopically and microcosmically. With the advancement of computer-aided image analysis technology, 3D reconstruction techniques have been useful for the study of peripheral nerves [1–6]. The 3D peripheral nerve visualization system can not only directly display the structures of peripheral nerves and the blood supply, but also reveal the branching and confluence of nerve fibers of different properties [7]. In addition, it can contribute to the analysis of the biological properties of peripheral nerves, and help in exploring the causes of functional abnormalities due to diseases and trauma [8], guiding the construction of ideal artificial nerves by tissue engineering and selecting the optimal therapeutic regimens.

At present, 3D peripheral nerve reconstruction is mainly based upon acetylcholinesterase histochemical method [9–11]. The main procedure includes preparation of specimens, acetylcholinesterase staining, 2D image acquisition, 2D image processing and 3D reconstruction. In the whole process of 3D reconstruction, the acetylcholinesterase staining procedure is a fundamental step for the success of 3D reconstruction. Detailed layer information of internal structures and textural properties in the section images are the basis of image processing and the key information ensuring the precision of 3D nerve reconstruction. So far, acetylcholinesterase staining is the main approach to distinguish the nerve fibers of different functions [9, 10, 12, 13] due to the convenient staining procedures, stable stained products, and the consistent textural property for the structures with an identical function. Currently, Karnovsky–Roots staining [9–13] is the only acetylcholinesterase staining method for 3D peripheral nerve reconstruction. However, the texture characteristics of the internal structure of the nerve are determined by the distribution characteristics of the acetylcholinesterase-positive sites. Karnovsky–Roots stained images cannot be automatically recognized by computer algorithm; and its image recognition totally depends upon manual image partition by specialists [11, 14, 15]. Currently, 3D reconstruction is often performed on the basis of acetylcholinesterase staining (which is believed to have a high quantity of sections) [16–18] and image partition of region of interest (ROI) is manually conducted on each stained image [19]. As such, the workload is extremely heavy and the accuracy of 3D reconstruction is not guaranteed. Taken together, Karnovsky–Roots acetylcholinesterase staining (that is currently employed for 3D nerve reconstruction) has several limitations [20, 21], such as arduous workload for image partition and low accuracy in 3D reconstruction outcomes [9].

Ideally, 2D staining section images should explicitly display nerve fascicles and nerve fiber functions with clear textures, which facilitate subsequent recognition, partition and 3D reconstruction of the section images [22, 23]. Currently, Karnovsky–Roots staining is the commonly employed staining method. However, this method fails to explicitly reflect the nerve fascicle regions and motor and sensory nerve fiber textures, which makes section image recognition and partition difficult, leading to a complicated 3D reconstruction and resulting in rough or inaccurate models [6, 20]. Therefore, a modified staining method is urgently required to enhance the display quality of textural property of ROI in stained images.

In this study, an acetylcholinesterase staining method for 3D peripheral nerve reconstruction was established based upon the principle of conventional Karnovsky–Roots staining, aiming to improve the display ability of nerve fascicle area and textures of motor and sensory nerve fibers. First, Karnovsky–Roots staining was conducted, and subsequently toluidine blue counterstaining was performed, followed by ponceau 2R counterstaining. This K-B-2R staining procedure was found to be able to better display the microstructure of myelin sheath, enhance the textural property of ROI, elevate the degree of recognition of section images and facilitate image partition and 3D nerve reconstruction and 3D printing.

## Materials and methods

The procedures of the current method can be described as the following steps. ① Sample preparation of peripheral nerve sections; ② Acetylcholinesterase staining, including Karnovsky–Roots staining, K-toluidine blue (K-B) counterstaining, and K-B-2R counterstaining; ③ 2D image acquisition, including image recognition, image mergence and image partition; ④ 2D image processing consisting of edge acquisition and functional recognition; ⑤ 3D reconstruction based on 2D images; ⑥ 3D printing based on standard triangulated language (STL) data of the 3D nerve model.

### Preparation of the peripheral nerve sections

Fresh right median nerve in the hand-wrist region was dissected from an adult cadaver with informed consent from the family. The specimen was approximately 15 mm in length and 3.4 mm in width and 1.5 mm in thickness at the proximal end, and 4.0 mm in width and 1.4 mm in thickness at the distal end. The nerves are extruded into an elliptical structure in this region due to the narrow space. Width and thickness were used to show the longer axis and shorter axis in the cross section, respectively. In addition, thickness was used to describe the distance between the superior and inferior planes of the nerve specimen. The epineurium and connective tissues of the median nerve specimen were removed and the specimen was placed in gradient sucrose solutions overnight at 4 °C. The obtained nerve specimen was fixed on a small piece of soft wooden board by pins to prevent the nerve from curling and to maintain it straight, embedded in OCT (optimal cutting temperature) cryostat embedding medium and quickly frozen at − 80 °C. The nerve specimen was cut into 5-mm segments and then sliced in a cryostat microtome (Tissue-Tek Cryo, Torrace, CA, USA) to obtain serial sections of 6 μm in thickness at − 24 °C. Thirty integral sections were selected and stained under the same condition.

### Acetylcholinesterase staining: K-B-2R staining

K-B-2R staining was divided into three procedures: (1) Karnovsky–Roots staining; (2) K-toluidine blue (K-B) counterstaining and (3) ponceau 2R counterstaining. After each staining procedure, image acquisition was carried out under light microscopy. Three staining groups of images were obtained, namely, Karnovsky–Roots, K-B, and K-B-2R staining groups. Karnovsky–Roots staining is a histochemical method for staining acetylcholinesterase. The method can qualitatively position nerve fibers through distribution characteristics of acetylcholinesterase-positive sites and can be used to distinguish the functional properties of nerve fibers to further guide clinical nerve repair. K-B is also a histochemical staining method that can stain myelin sheath of myelinated nerve fibers to show the myelin sheath structure. This staining method can be used to observe the myelin sheath structure under an electron microscope. K-B-2R is a staining method for showing myelin sheath of myelinated nerve fibers. After staining, the morphology of myelin sheath can be displayed, and K-B-2R method can be used in pathological diagnosis and research.

*Karnovsky*–*Roots staining*: Firstly, the staining solution was prepared 20 min before staining. The incubation medium was prepared as follows: 12.5 mg butyrylthiocholine used as the substrate was dissolved in 16 ml of 0.1 M phosphate buffer. Then 1 ml of 0.1 M sodium citrate, 2.5 ml of 0.5 M copper sulfate solution, 2 ml distilled water, and 2.5 ml of 0.05 M potassium ferricyanide were mixed with stirring. The resultant incubation solution should be clear, light green and transparent liquid. The specimen sections were completely immersed in the staining solution at 4 °C for 24 h, followed by rinse in distilled water, dehydration in graded ethanol solutions and section mounting in neutral balsam. Once stained and mounted, Karnovsky–Roots stained sections were immediately partitioned and photographed, and 30 images were acquired.

*K*-*B staining*: Karnovsky–Roots stained sections were firstly immersed in the xylene to remove the cover glass and neutral balsam. Solution was prepared as follows: 1 g toluidine blue and 1 g sodium borate were added with 100 ml distilled water and dissolved completely. Then, the sections were immersed in 1% toluidine blue solution and incubated in water bath at 37 °C for 15 min. After staining, the stained sections were rinsed in distilled water, dehydrated in graded ethanol solutions and mounted in neutral balsam. Then, K-B stained sections were immediately partitioned and photographed, and 30 images were acquired.

*K*-*B*-*2R staining*: Firstly, K-B stained sections were immersed in xylene to remove cover glass and neutral balsam. Solution was prepared as follows: 1 g ponceau 2R and 2.5 ml glacial acetic acid added with 100 ml distilled water and dissolved completely. Then, ponceau 2R counterstain was performed at 25 °C for 5 min, 1% phosphotungstic acid for differentiation, immersed in 1% glacial acetic acid for 10 s, rinsed in distilled water, dehydrated in graded ethanol solutions and mounted in neutral balsam. Eventually, ponceau 2R stained sections were partitioned and photographed, and 30 partitioned images were acquired.

### Image acquisition

Thirty stained sections were observed under light microscope (× 100) to explicitly display the nerve fascicles, acetylcholinesterase-positive sites and myelin sheath. As the specimen of median nerve could not be fully observed within one visual field, the sections should be partitioned and then merged to acquire the full view cross-section image. During image partition and photography, each section of every staining step was partitioned into 12 regions with one partition within one visual field. The image photography began from the left top corner of each section and continued row by row. The images saved as JPEG files were labeled as 1–12.

The Eclipse 50i light microscope (Nikon, Tokyo, Japan) equipped with Power shot S70 digital camera (Canon, Tokyo, Japan) was used for image acquisition, with various parameter set as below: automatic white balance off, ISO value 200, aperture 8, focal length 5.813 mm, exposure compensation 0, 3072 × 2304 pixel in size, resolution of 180 dpi.

Image acquisition consisted of three procedures, namely image recognition, image mergence and image partition. Firstly, image recognition was to recognize the textural properties of 2D images of all 12 partitions of each staining step. Then, the images were entered into the PTGui Pro 8.3.7 panorama software (New House Internet Services BV, Rotterdam, Holland). Image mergence was to merge different partitioned images into one single image by the alignment of the 12 2D partitioned images to construct a seamless, high-resolution one full panorama cross-section image of the nerve. Adjacent images with identical textural properties could be merged with > 15% overlap. Compared with individual partitioned images, the panorama image had a higher resolution and contained more intact nerve information. Lastly, image partition was to divide the panorama image into a series of uniform, non-overlap partitions to obtain the contours of nerve fascicle regions of different layers for subsequent edge acquisition and functional recognition step.

### Image processing

Image processing was divided into two stages, i.e. edge acquisition and functional recognition. More details about algorithm of image processing were reported in our previous study [24]. The nerve fascicle edges were acquired after obtaining the central positions of nerve fascicles, the number of nerve fascicles and pixel groups of nerve fascicles. The edges for the nerve fascicles were smoothed [24]. An unsupervised dynamic clustering method was implemented to obtain the accurate edges of nerve fascicular because the number of nerve fascicular varied due to converge and split of nerve fascicular. A reasonable classification algorithm was used to obtain automatic functional recognition for nerve fascicles. The features of the pixel neighborhood in nerve slice images were represented using the second-order gradient and multi-directional gradient method.

## 3D reconstruction

The 2D nerve fascicle contours of nerve sections were obtained and labelled with numbers according to the sequence of section preparation. Then the 3D nerve models were reconstructed using a 3D reconstruction software package Mimics (Materialise, Leuven, Belgium) based on obtained the 2D images. An appropriate mask was created for each nerve fascicle and the mask had to cover the whole cross-section of each nerve fascicle. In addition, to identify every nerve fascicle in one nerve group, the different colors were selected to represent various nerve fascicles.

## 3D printing

3D printing was performed by using the Raise3D N2 Plus rapid prototyping machine (Raise3D Inc., Shanghai, China. Process of fused deposition modeling (FDM) and the raw material of polylactic acid (PLA) were used in 3D printing. Molding temperature and squeeze speed were set as 215 °C and 10 mm/s, respectively. Then, the STL format data of 3D nerve model was imported to the rapid prototyping machine, the output sample was scaled in proportion of 1:10, and the layer thickness was 0.3 µm. Finally, a cylindrical base was added to the 3D model of the nerve bundle.

### Validating experiments with other nerve specimens

The reproducibility test of K-B-2R staining consisted of staining of four other nerve specimens obtained, including another segment of the median nerve, and segments of nerves of different functions and properties namely distal nerve fascicle of the median nerve, muscle nerve branch and sural nerve. After K-B-2R staining, the sections were partitioned, photographed and merged into the panorama images. Eventually, the panorama images were subjected to image partition, image recognition and 3D reconstruction as described above.

*Another median nerve*: For obtaining another segment of median nerve, a fresh right median nerve specimen was dissected from the right hand-wrist region (approximately 5 mm in length, 3.3 mm in width and 1.4 mm in thickness at the proximal end, and 3.5 mm in width and 1.3 mm in thickness at the distal end). The specimen was continuously sectioned and 240 intact sections were used for the test procedure.

*Mixed, motor and sensory nerve fascicles*: To obtain segments of nerves of different functions and properties, the distal segments of the distal nerve fascicles were dissected from the median nerve (approximately 1 mm in length, 0.3 mm in width, and 0.2 mm in thickness), muscle nerve branch (0.2 mm in length and approximately 0.1 mm in diameter) and sural nerve (approximately 1 mm in length and 0.5 mm in diameter). Respectively from these nerve segments, 300, 60 and 60 intact sections were selected for the staining reproducibility test procedure.

### Evaluation of the staining quality

*Nerve fascicle regions*: Acetylcholinesterase-positive sites represent the texture of nerve fibers of different functions and properties, and these positive sites form the nerve fascicle regions. Since the goal of acetylcholinesterase staining was to enhance the display quality of nerve fascicles and nerve fiber textural properties without affecting the distribution and the quantity of acetylcholinesterase-positive sites, evaluation of the staining quality was necessary.

*Quantitative analyses*: Image Pro Plus 6.0 software (Media Cybernetics, Bethesda, Maryland, USA) and SPSS 19.0 statistical software (IBM, Armonk, New York, USA) were used for the staining quality evaluation. The counting of acetylcholinesterase staining sites was performed using Image Pro Plus 6.0 software. The full view image data were analyzed using SPSS 19.0 statistical software. All data were expressed as mean ± standard deviation. Group comparison was conducted by a mixed linear model, and *α *= 0.05 was considered as statistically significant.

## Results

### Image acquisition

**Fig. 1:**
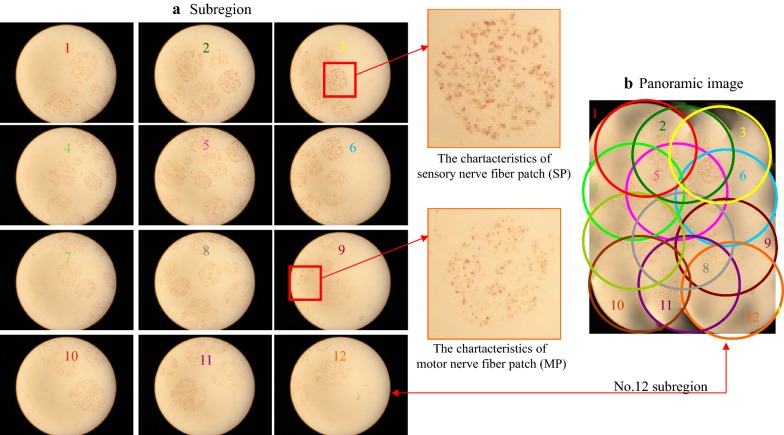
The 12 sub-region images of a median nerve section (No. 20) following Karnovsky–Roots staining and their mergence to create the panoramic image, **a** sub-region and **b** panoramic image

One section (No. 20) was randomly selected from 30 sections for the illustration of the K-B-2R staining results. Figure 1 shows the 12 partitioned images and panorama image of No. 20 section after Karnovsky–Roots staining. Brownish-yellow spots were evident in the partitioned images, forming a circle-like region without excessive color among different spots (Fig. 1a). In the panorama image, the edge of each nerve fascicle region was not explicit (Fig. 1b). Partitioned images 3 and 9 revealed the characteristics of acetylcholinesterase-positive sites in the aggregation regions of nerve fibers of different functions and properties. Image 3 shows the typical spots in the aggregation regions of sensory nerve fibers. The feature of sensory nerve fiber is a dark brownish-yellow spots with a large area and irregular shape, and located in the nerve bundle region. Image 9 reveals the typical spots in the aggregation regions of motor nerve fibers. Motor nerve fibers are elliptical, with light brownish-yellow uniform spots, and are located within the nerve bundles.

**Fig. 2:**
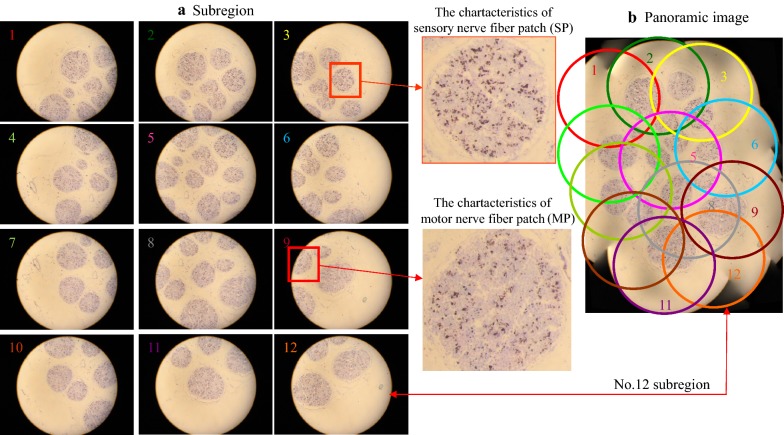
The 12 sub-region images of a median nerve section (No. 20) following K-B staining and their mergence to create the panoramic images, **a** sub-region and **b** panoramic image

Figure 2 shows the 12 partitioned images (Fig. 2a) and panorama image (Fig. 2b) of No. 20 section after K-B staining. In the partitioned images, nerve fascicles formed circle-like regions with explicit edges, and blue black spots were observed with light blue excessive color (Fig. 2a). The aggregation regions of sensory nerves were lavender in color with irregular blue black spots with a relatively large size, as shown in the partitioned image 3. Partitioned images 3 and 9 revealed the characteristics of acetylcholinesterase-positive sites in the aggregation regions of nerve fibers of different functions and properties, and the typical spots of the motor and sensory nerve fibers were identical to the results of K-R staining (Fig. 2). Motor nerve fibers are distributed within the nerve fibers. Sensory nerve fibers have dark brownish-yellow spots of large areas and of irregular shapes, and are located in the nerve bundle region.

**Fig. 3:**
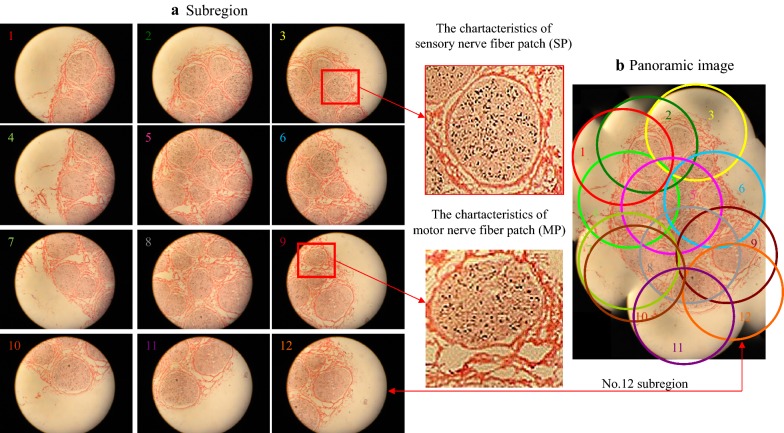
The 12 sub-region images of a median nerve section (No. 20) following K-B-2R staining and their mergence to create the panoramic image, **a** sub-region and **b** panoramic image

Figure 3 shows the 12 partitioned images (Fig. 3a) and panorama image (Fig. 3b) of No. 20 section after ponceau 2R counterstain. In the partitioned and panorama images, the connective tissues in the nerve specimen were pink in color, nerve fascicle regions were stained lightly red with explicit edges, and acetylcholinesterase-positive sites were blackly counterstained spots, with pale red transitional regions being observed between spots (Fig. 3). Partitioned image 3 revealed the typical spots of sensory nerve fibers. Partitioned image 9 revealed the typical spots of motor nerve fibers, which were consistent with the distribution characteristics of Karnovsky–Roots (Fig. 1) and K-B staining (Fig. 2). The acetylcholinesterase-positive sites were distributed in the middle of the sensory nerve fibers, i.e., sensory nerve fibers were distributed among the acetylcholinesterase-positive sites. Among the nerve fibers, the characteristic spots of motor nerve fibers were located in the annular myelin sheath, and the characteristic spots of sensory nerves were located between the annular myelin sheaths.

**Fig. 4:**
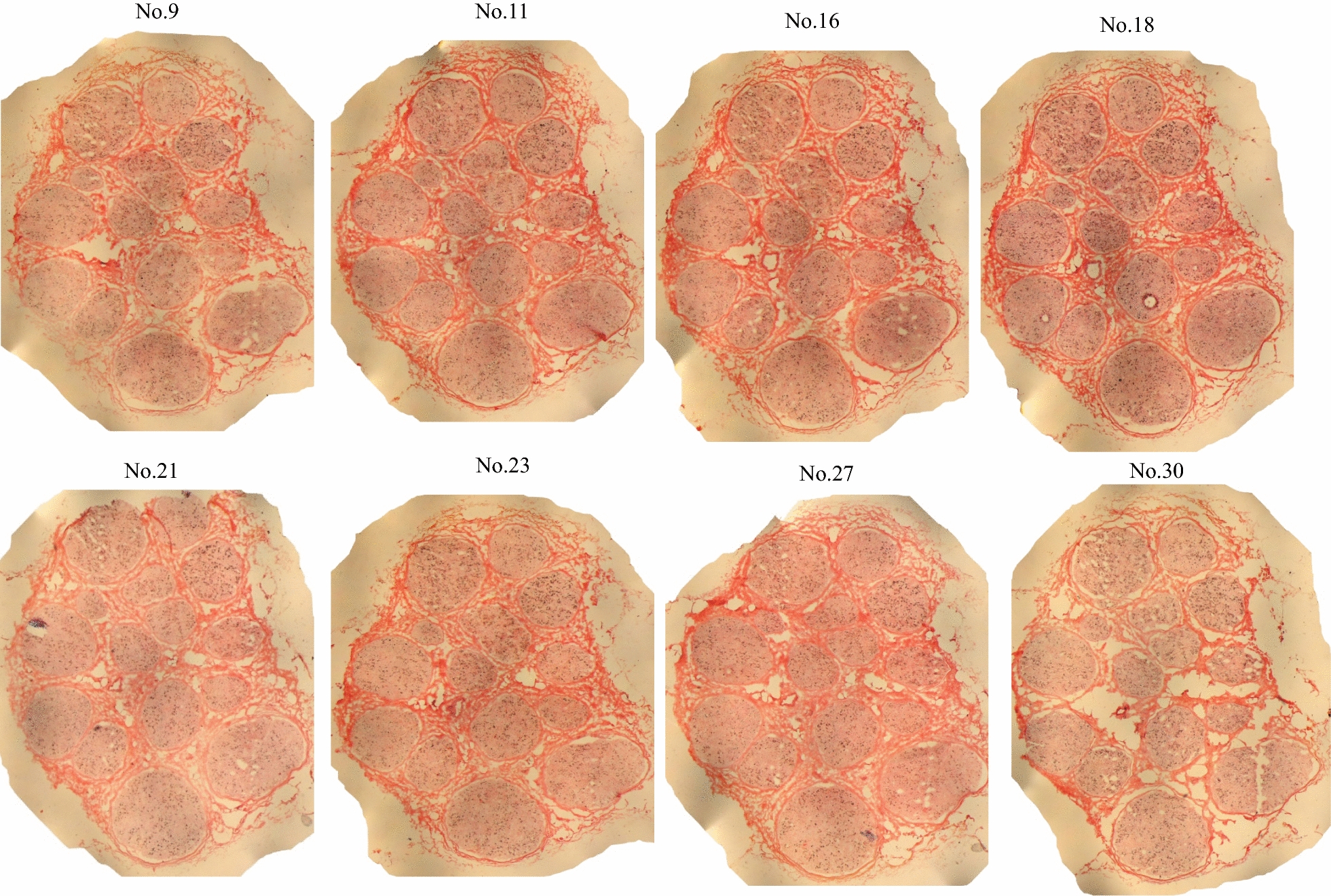
Image acquisition of a series of the median nerve

The median nerve specimen was continuously sectioned with 6 μm in thickness and a series of sections were subjected to K-B-2R staining. Partial results of the section staining were illustrated in Fig. 4 (staining of Nos. 9, 11, 16, 18, 21, 23, 27 and 30 sections). The contours of all nerve cross sections were almost consistent. The epineurial structures were clearly displayed in the staining sections. Among 30 specimen sections, three sections (Nos. 2, 3 and 29 sections) were severely destroyed after K-B-2R staining. The nerve fascicle structure in the remaining 27 intact sections was almost intact, and only some minor tissue defects were observed among nerve fascicles in some of these sections.

### Edge acquisition

**Fig. 5:**
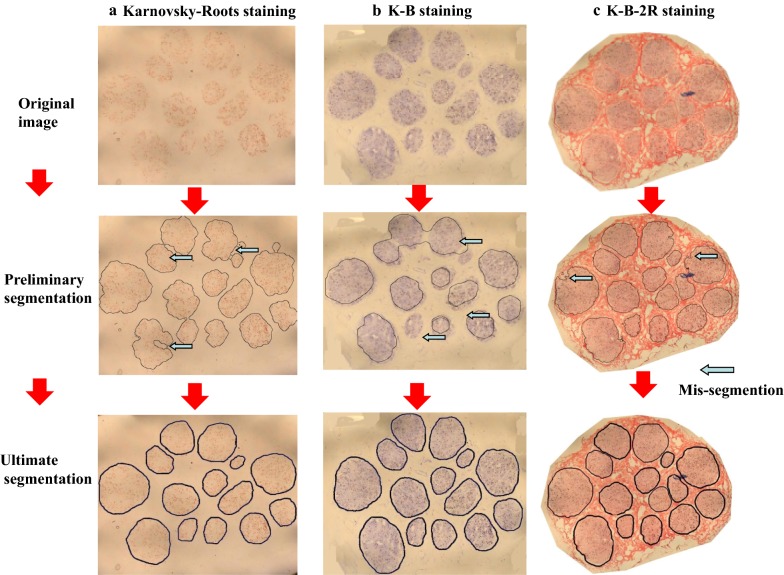
Edge acquisition of nerve fascicles in median nerve sections (No. 20) after different staining methods. **a** Karnovsky–Roots staining; **b** K-B staining; and **c** K-B-2R staining

**Fig. 6:**
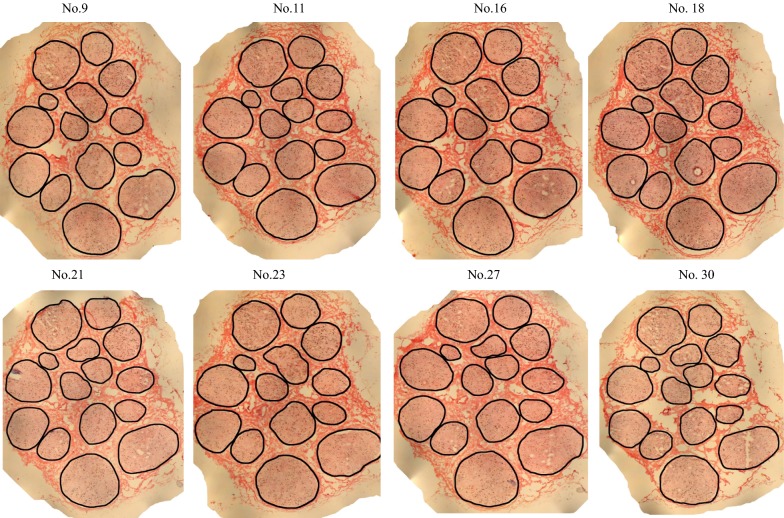
Edge acquisition of a series of median nerve sections

The results of image partition are illustrated in Fig. 5. As shown, Karnovsky–Roots staining caused the image acquisition of nerve fascicle edges being significantly different from the actual nerve region with multiple wrong partitions (Fig. 5a). After K-B staining, the consistency between the nerve fascicle of partitioned image and the actual nerve fascicle contour was enhanced. However, the non-specific staining among nerve fascicles also led to frequent occurrence of wrong partitions (Fig. 5b). After K-B-2R staining, however, the nerve fascicle contour was basically consistent with the actual nerve fascicle contour. The selected images contained some contaminant spots (blue black spots among the nerve fascicles were contaminant deposition in the specimen). In the preliminary acquisition image, no contaminants were observed which might cause wrong partition (Fig. 5c). The results of the acquisition of the nerve fascicle edges in the median nerve sections (images Nos. 9, 11, 16, 18, 21, 23, 27 and 30 sections) are shown in Fig. 6.

### Functional recognition

**Fig. 7:**
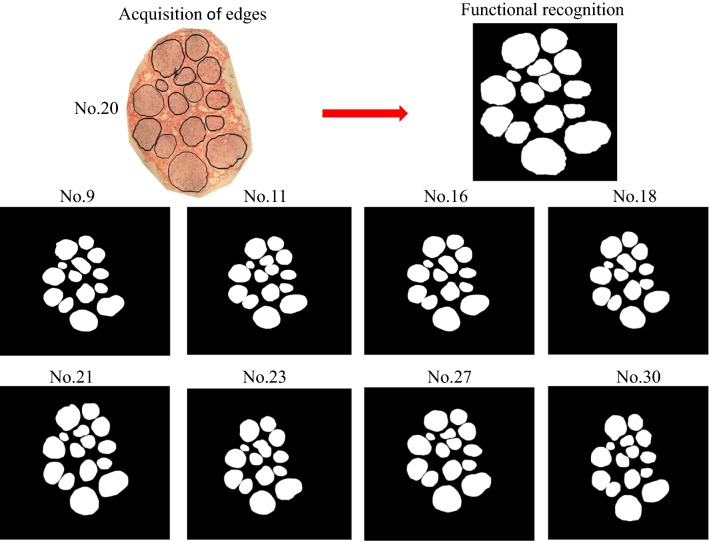
Functional recognition of nerve fascicles

After the edge acquisition of a series of sections in the median nerve sections, functional recognition of nerve fascicle in median nerve was implemented. The results of the functional recognition of the nerve fascicle in the median nerve (as shown in images Nos. 9, 11, 16, 18, 21, 23, 27 and 30) are presented in Fig. 7.

## 3D reconstruction

**Fig. 8:**
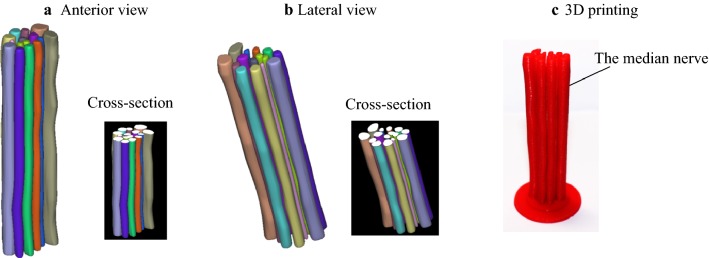
3D nerve reconstruction and 3D printing of the median nerve. **a** Anterior view, **b** Lateral view and **c** 3D printing model

Figure 8 illustrates the results of the 3D nerve reconstruction of the median nerve according to the corresponding functional recognitions of the nerve fascicles. It can be observed that the numbers and the shapes of the median nerve fascicles are the same between section No. 21 (in Fig. 7) and the reconstructed image (Fig. 8b). However, from Fig. 8, it can be found that the numbers of nerve fascicles are different for the different sections, and this is because some nerve fascicles split and some converge at the different sections.

## 3D printing

Based on the above results of the 3D nerve reconstruction and the 3D geometrical data in the standard triangulated language (STL) format of the median nerve, the corresponding 3D model of the median nerve was produced by using a 3D printing machine (Raise3D N2 Plus rapid prototyping machine) (Fig. 8c). To present the location of each nerve fascicle, one end of all the nerve fascicles was attached to a circular disk. The 3D shape of the median nerve can be clearly observed (Fig. 8c), which enables clear and easy understanding of peripheral nerve structure.

### Validating experiments with other nerve specimens

**Fig. 9:**
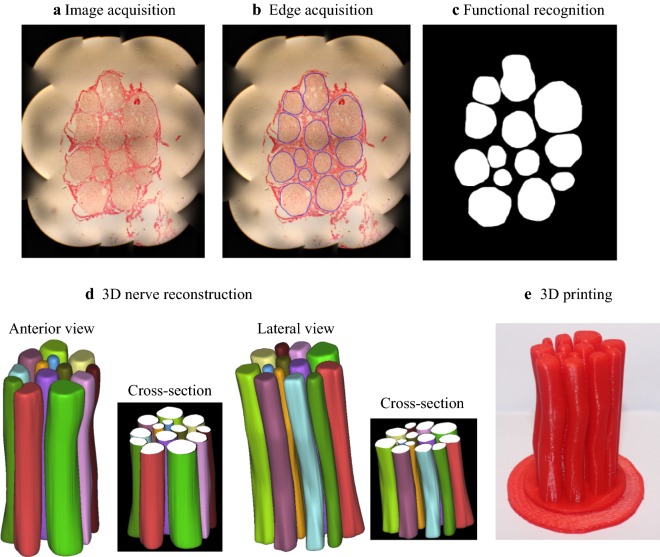
Staining, functional recognition, 3D reconstruction and 3D printing results of another segment of the median nerve (No. 40) after K-B-2R staining. **a** Image acquisition; **b** edge acquisition; **c** functional recognition; **d** 3D nerve reconstruction (front view and lateral view); and **e** 3D printing

In order to validate the effectiveness of the proposal method, the reproducibility test was conducted with another group of median nerves. Results of the image acquisition, edge acquisition after K-B-2R staining and functional recognition of one segment of the median nerve (No. 40) are shown in Fig. 9a–c, respectively. The 3D reconstruction and 3D printing results of the different segments of the median nerve are presented in Fig. 9d, e. It can be clearly seen that the numbers and the shapes of nerve fascicles of the median nerve in Fig. 9a–c are coincident with those in Fig. 9d, e.

**Fig. 10:**
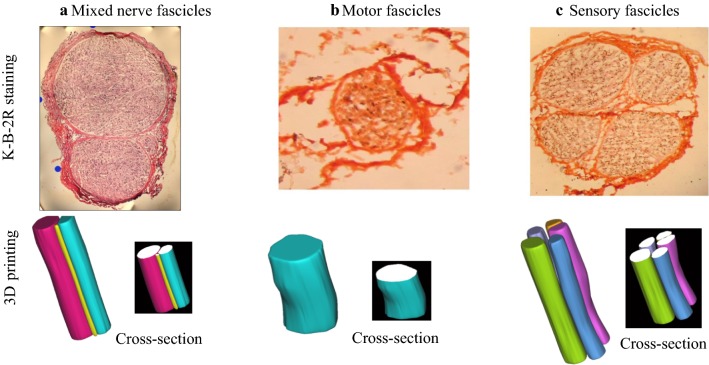
K-B-2R staining results of the mixed nerve fascicles. **a** Mixed nerve fascicles (No. 20); **b** motor fascicles (No. 9); **c** Sensory fascicles (No. 1) and 3D nerve reconstruction of mixed nerve fascicles

Figure 10a demonstrates a section of the distal branch of the hand-wrist median nerve which contains the mixed nerve fascicles. The nerve fascicles consisted of typical spots of motor and sensory nerve fibers. The perineurium and epineurium were explicitly displayed. From Fig. 10b, it can be seen that a section of the nerve branch dominating the hand muscle which contains only motor nerve fascicles. The nerve fascicle mainly consisted of typical spot aggregations of the nerve fibers. In addition, in Fig. 9c, a section of sural nerve is a sensory nerve, with all nerve fascicles containing typical spots of sensory nerve fibers. Acetylcholinesterase-positive sites were sporadically distributed among the ring-shaped myelin sheath structures.

The 3D reconstruction was implemented according to a series of images after functional recognition. In Fig. 10d, three nerve fibers are shown, i.e. motor fascicles (yellow and red) and sensory nerve fascicle (cyan). In Fig. 9, only one nerve fascicle (motor fascicle) is presented. From Fig. 10d, it can be seen that five nerve fibers are reconstructed, and all the never fascicles are sensory fibers.

### Evaluation of the staining quality

**Fig. 11:**
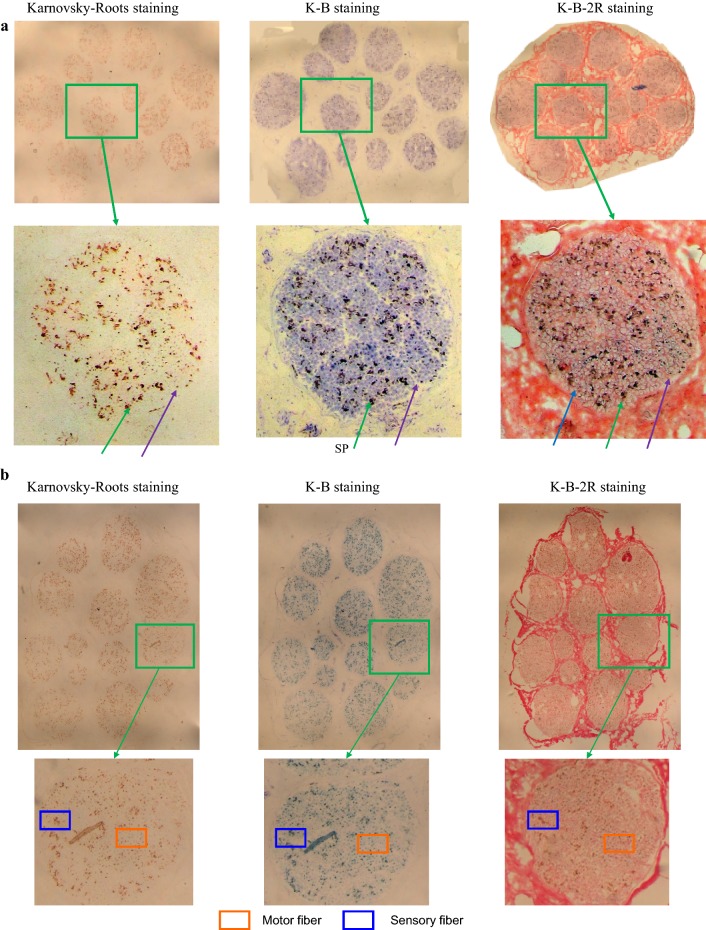
Textural properties of nerve fascicles and nerve fibers of different functions and properties of median nerve after different methods of staining (SP-sensory nerve fiber patch; MP-motor nerve fiber patch). A segment of median nerve (No. 20) and **b** a segment of median nerve (No. 40)

For direct comparisons, panorama images of the same specimen after Karnovsky–Roots, K-B and K-B-2R staining are shown in Fig. 11a. The green frame in each panorama image indicates the nerve fascicle at the same site. After three steps of staining, no changes were observed in the distribution of nerve fascicles at the same positions of the same specimen. The distributions of acetylcholinesterase-positive spots were also consistent in the nerve fascicles, with the typical spots in the nerve fibers of different functions and properties remaining unchanged.

After staining, 226 sections were intact with consistent staining characteristics. Textural properties of nerve fascicles and nerve fibers of different functions and properties of median nerve after different steps of staining are presented in Fig. 11b for Karnovsky–Roots staining, K-B staining and K-B-2R staining, respectively. Karnovsky–Roots staining revealed nerve fascicle regions consisting of brown spots, with the nerve fascicles and nerve edges not being clearly displayed and the brown spots being formed from the remnant pigment sediment (Fig. 11b). For K-B staining, discontinuous perineurial and epineurial structures were observed at the edges of nerve fascicles and nerve. Light blue staining as the transitional region was observed in between the typical spots of the nerve fibers of different properties; however, myelin sheath edges were indistinct (Fig. 11b). The connective tissues in between the round nerve fascicles were evenly stained as pink/red for K-B-2R staining (Fig. 11b).

Furthermore, acquisition of nerve fascicle edges among the K-B-2R stained sections consumed the minimal time and required the minimal manual intervention. Table 1 shows the comparisons of the acquisition processes for nerve fascicle edges of the median nerve section panorama images resulting from different staining methods.

**Table 1:** Comparison of internal structure textures of the median nerve sections following different staining methods

Group	Karnovsky–Roots staining	K-B staining	K-B-2R staining
Nerve fascicle texture	Aggregation region of diffuse spots	Pale blue circle-like region	Reddish circle-like region
Nerve fascicle edge	No	Indistinct	Clear
Space among nerve fascicles	Slight non-specific staining	Intermittent non-specific staining	Even staining
Myelin sheath	No	No	Clear
Motor nerve fiber	Indistinct	Indistinct	Explicit
Sensory nerve fiber	Indistinct	Indistinct	Explicit
Relationship between myelin sheath and typical spots of nerve fibers	–	–	Explicit
Acetylcholinesterase-positive site	Brownish-yellow	Blue black	Black
Transitional region among spots	Occasional non-specific staining	Even pale blue	Reddish

The numbers of acetylcholinesterase-positive sites were (21.63 ± 4.06) × 10^2^, (20.64 ± 3.51) × 10^2^ and (20.54 ± 5.71) × 10^2^ in the Karnovsky–Roots staining, K-B staining and K-B-2R staining groups, respectively, which suggest that quantities of acetylcholinesterase-positive sites did not significantly change after K-B-2R staining. Furthermore, the distribution characteristics of acetylcholinesterase-positive sites were consistent and no obvious differences were observed by the three staining methods.

## Discussion

3D reconstruction of peripheral nerves is one important tool to understand the nerve internal information and functional basis, and it is gradually becoming a hot spot of the peripheral nerve research field [3, 14, 15, 24–28]. 2D acetylcholinesterase staining is a key technique for 3D peripheral nerve reconstruction, which affects the precision and accuracy of the 3D reconstruction images. Ideal 2D staining section images are those that are able to explicitly display the nerve fascicles and the nerve fibers of different functions with clear textural properties, which can facilitate subsequent image recognition, partition and 3D reconstruction. At present, Karnovsky–Roots staining is the commonly employed 2D staining method. However, this staining technique can not explicitly display the nerve fascicles and textural properties of the motor and sensory nerve fibers. Consequently, using the resulting images, it is difficult to establish an accurate 3D model due to the poor effect of image recognition. We have recently explored the 3D reconstruction of the peripheral nerves based on traditional 2D staining method [12, 13, 24, 29]. Improving image texture features is still a difficult problem to solve. Myelin may be a useful structure. In theory, both sensory and motor nerve fibers are myelinated nerve fibers. If the myelin can be stained on the basis of Karnovsky–Roots staining method, the texture characteristics of the inner structure of the nerve may be improved. There are some methods to show myelin, for example, toluidine blue and Ponceau 2R have been used as effective staining methods for the study of myelin morphology in research and clinical work [30]. While these staining methods offer more options for improving image quality, no one was used in research of 3D reconstruction. The use of two staining methods either separately or together based on Karnovsky–Roots staining method to show the myelin sheath has not been reported in the current literature. This study found that counterstaining can improve the image quality. In the current study, the K-B-2R staining method was developed by modifying the conventional acetylcholinesterase staining. This K-B-2R new staining method has been shown to be able to display the axon and myelin sheath simultaneously, which significantly improves the display quality of the nerve fascicle regions, and the motor and the sensory nerve fiber textures. The resulting images of the peripheral nerve have led to a high degree of recognition, image partition and accomplishment of the 3D reconstruction (Figs. 8, 9d and 10d). Importantly, the reconstruction cycle was enormously shortened and the precision of 3D reconstruction model was significantly enhanced. In addition, the 3D printing technology was applied to create the 3D digital model of nerve fascicle (Figs. 8c and 9e). Thus, this new staining technique can facilitate 3D reconstruction and creation of the 3D digital model, which suggests that this new technique can facilitate to rebuild and repair the nerve fascicles when it is used in conjunction with the 3D reconstruction and 3D printing technologies.

Traditional staining method of peripheral nerve sections has several limitations [9, 25]. Firstly, as the sections of nerve specimens are several microns in thickness, it is difficult to successfully prepare adjacent sections in a large number, which causes missing data and reduces the accuracy of images. Secondly, the section’s partial loss during the staining process is different for each section, and the accumulation of loss error during image mergence is likely to lead to distortion of the merged image.

Our previous works [12, 13, 29] have demonstrated that the simultaneous display of both axon and myelin sheath of motor and sensory nerves can avoid the incidence of such errors during image mergence. While the axons and myelin sheaths of motor and sensory nerves are the common parameters for observation and analyses of the physiological pathological states of nerve fibers, various reagents and equipment can display the morphology of axons and myelin sheaths. As the myelin sheath can be explicitly displayed by toluidine blue and ponceau 2R staining, 2D K-B-2R staining method was proposed in the study. The functions, properties and distributions of the nerve fibers can be determined by the morphology of both axon and myelin sheath.

In the current study, 2D K-B-2R staining was found to be able to determine the properties and distributions of nerve fibers. Acetylcholinesterase-positive sites are the unique signs of Karnovsky–Roots staining as also shown in the current study (Fig. 5a), and the changes in the distribution characteristics or quantities of acetylcholinesterase-positive sites might affect the precision of 3D reconstruction. Herein, no evident changes were documented in the distribution characteristics or quantities of acetylcholinesterase-positive sites after K-B-2R staining (Fig. 5c), with the results of having the same positions of the positive sites on the identical specimen section as with Karnovsky–Roots staining. In addition, no statistically significant differences were observed in the quantities of acetylcholinesterase-positive sites identified by the three staining methods. Furthermore, during K-B-2R staining, the myelin sheath staining contributed to the exclusion of the possibility of non-specific dye sediment (Table 1). These results suggest that K-B-2R staining does not significantly affect the Karnovsky–Roots staining results, but it can additionally determine the properties and the distribution sites of the nerve fibers.

In addition, K-B-2R staining is favorable for the subsequent section image stitching step of 3D reconstruction. In Karnovsky–Roots stained images, the staining color is plain. The brown spots formed by acetylcholinesterase-positive sites (Figs. 1 and 5a) are the primary texture signs for image mergence. While approximately 20–30% of overlap is required between adjacent images, even 50% overlap is sometimes required for certain regions. As illustrated in Figs. 3 and 5c and Table 1, K-B-2R staining could explicitly display the nerve fascicle regions and the myelin sheath structures of the nerve fibers. The connective tissues were obviously stained with clear edges, which could be used for image mergence with abundant textures, allowing adjacent images with 15–20% overlap being merged into a panorama image. Due to this connective tissue staining, the K-B-2R staining method is advantageous for nerve specimens with large cross section areas. It not only significantly decreases the frequency of image partition and photography, reduces the workload of image mergence, enhances working efficiency, but also minimizes the accumulated error of manual operation and enhances the precision of image mergence results.

In the current study, the 2D K-B-2R staining method was found to be more suitable for image partition of nerve fascicle regions. Comparisons of the automatic image partition of the nerve fascicle regions from each of the three methods (as performed using the same algorithm method) (Fig. 5) show that K-B-2R staining obviously has significant advantages in displaying nerve textures. Firstly, by comparing the acquired nerve fascicle edges and the actual edges, the acquired nerve fascicle edges revealed in Fig. 5 in Karnovsky–Roots stained images were inconsistent with the actual edges, which required manual processing (Fig. 5a). Compared with Karnovsky–Roots stained images, the nerve fascicle edges acquired in K-B stained images were more satisfied; however, they still required manual processing and adjustment, and the non-specific staining among nerve fascicles was increased, leading to more frequent wrong partition. On the other hand, the nerve fascicle edges acquired in K-B-2R stained images were the most consistent with the actual situations. Since the texture properties of nerve fascicle regions and nerve fascicles significantly differed, despite the existence of some non-specifically stained spots, the probability of wrong image partition was enormously reduced in K-B-2R stained images.

Furthermore, the obtained images by this method required less time for slight manual processing and adjustment (Fig. 5). In Karnovsky–Roots and K-B stained images, evidently wrong partitioning occurred in the acquired nerve fascicle edges, the operation time was long (Table 1), and the partitioning required multiple manual interventions. In contrast, the partition results obtained by the K-B-2R staining were almost identical to the actual nerve fascicle edges, which required few manual interventions and shorter operation time. Thus, the 2D K-B-2R staining method proposed in the current study is more advantageous for image recognition compared with alternative approaches.

The current study shows that the K-B-2R staining method enabled staining of the connective tissues within the nerves especially the nerve fascicle regions, revealing the ring-shaped perineurial structures (Fig. 5c), which significantly contribute to images of nerve fascicle regions. In addition, the myelin sheath edges were explicitly displayed with this staining method, with each myelin sheath corresponding to each nerve fiber, and with a large quantity of myelin sheath edges aggregating in the nerve fascicle regions. These features significantly differed from those of the connective tissues among nerve fascicles. Among the axons wrapped by myelin sheaths, the motor nerve fibers were stained, whereas the sensory nerve fibers were not stained. Hence, with this staining method, it is more convenient to distinguish the nerve fascicles and the nerve fibers of different functions and properties, which contributes to image partition.

Data from the current study suggest that the 2D K-B-2R staining method is applicable for batch staining. Reconstructing and displaying the intact peripheral nervous system will require preparing and staining of an extremely large quantity of sections of several microns in thickness without compromising efficiency and quality. The 2D K-B-2R staining method established in the current study has been shown to cause little specimen loss and yet produce stable staining outcomes. Among the 30 sections of median nerve, 27 intact sections were stained and shown to have a consistent textural property (Fig. 11a). In the repeated experiments, among the 240 median nerve sections stained, 226 sections were found intact with a consistent textural property (Fig. 11b). Respectively from the total 300 sections of distal nerve fascicles of the median nerve, 60 sections of the muscle nerve branch, and 60 sections for the sural nerve, 268, 63 and 61 intact sections were obtained with a complete data set following K-B-2R staining. These data suggest a low section loss/damage rate and a high success rate with a complete data set being obtained by the staining method.

The current study compared the staining qualities among the three different methods. Karnovsky–Roots stained sections revealed the nerve fascicle regions being circle-like structures consisting of brown spots, and with no explicit and continuous nerve fascicle membrane structures being noted. A slight quantity of connective tissues among nerve fascicles was stained (Fig. 11a). No transitional color was noted in between the aggregation regions of sensory and motor nerve fibers and among the typical spots of nerve fibers of different properties (Fig. 11b). After K-B staining, nerve fascicle regions were in pale blue color, with nerve fascicle membrane structure being revealed. However, non-specific staining of the connective tissues among nerve fascicles was increased (Fig. 11a). K-B-2R staining revealed clear nerve fascicle membrane structures, and connective tissues among nerve fascicles were evenly stained and the myelin sheath of nerve fascicles was stained light red. The staining color of nerve fascicle regions and surrounding tissues significantly differed. The blue spots resulted from remnant pigment sediment (Fig. 11a). Discontinuous staining was noted in the connective tissues among nerve fascicles, epineurium and perineurium (Fig. 11a). However, the distribution characteristics of acetylcholinesterase-positive sites were consistent between K-B staining and Karnovsky–Roots staining. Perineurial and epineurial structures were seen at the edges of nerve fascicles and nerve. The acetylcholinesterase-positive sites were stained as black. The typical spots of motor nerve fibers were enveloped by the ring-shaped myelin sheath structures, and the typical spots of sensory nerve fibers were distributed in between the ring-shaped myelin sheath structures (Fig. 11b).

## Conclusion

In this study, based on the Karnovsky–Roots staining technique, a 2D staining method was established, which has significantly increased the display quality of nerve fascicle regions and fiber textures of both motor and sensory nerves. In this K-B-2R staining, Karnovsky–Roots staining was firstly performed, followed by toluidine blue counterstain, and then ponceau 2R counterstain. This technique can enable the display of both axons and myelin sheaths within one single section. Through this technique, the display quality of the nerve fascicles, motor and sensory nerve fiber textures was significantly enhanced, and peripheral nerve section images yielded a high degree of recognition, which was beneficial for subsequent image partition 3D reconstruction. This 2D K-B-2R staining method has significantly shortened the cycle and evidently enhanced the precision of 3D peripheral nerve reconstruction. This technique has thus appropriately resolved the technical challenge faced by nerve injury repair. Furthermore, the 3D reconstruction and 3D printing technology can provide an ideal solution for the nerve injury repair in the field of nerve tissue engineering if the appropriate printing material is applied.

## Data Availability

Please contact the corresponding author for data on reasonable request.

## Abbreviation

3D: three-dimensional
2D: two-dimensional
K-B-2R: Karnovsky–Roots toluidine blue ponceau 2R
ROI: region of interest
K-B: K-toluidine blue
STL: standard triangulated language
OCT: optimal cutting temperature
FDM: fused deposition modeling
PLA: polylactic acid
